# Inhibition of endothelial histone deacetylase 2 shifts endothelial-mesenchymal transitions in cerebral arteriovenous malformation models

**DOI:** 10.1172/JCI176758

**Published:** 2024-05-23

**Authors:** Yan Zhao, Xiuju Wu, Yang Yang, Li Zhang, Xinjiang Cai, Sydney Chen, Abigail Vera, Jaden Ji, Kristina I. Boström, Yucheng Yao

**Affiliations:** 1 Division of Cardiology, David Geffen School of Medicine at UCLA, Los Angeles, California, USA.; 2 The Molecular Biology Institute, UCLA, Los Angeles, California, USA.

**Keywords:** Vascular biology, Endothelial cells, Mouse models

## Abstract

Cerebral arteriovenous malformations (AVMs) are the most common vascular malformations worldwide and the leading cause of hemorrhagic strokes that may result in crippling neurological deficits. Here, using recently generated mouse models, we uncovered that cerebral endothelial cells (ECs) acquired mesenchymal markers and caused vascular malformations. Interestingly, we found that limiting endothelial histone deacetylase 2 (HDAC2) prevented cerebral ECs from undergoing mesenchymal differentiation and reduced cerebral AVMs. We found that endothelial expression of HDAC2 and enhancer of zeste homolog 1 (EZH1) was altered in cerebral AVMs. These alterations changed the abundance of H4K8ac and H3K27me in the genes regulating endothelial and mesenchymal differentiation, which caused the ECs to acquire mesenchymal characteristics and form AVMs. This investigation demonstrated that the induction of HDAC2 altered specific histone modifications, which resulted in mesenchymal characteristics in the ECs and cerebral AVMs. The results provide insight into the epigenetic impact on AVMs.

## Abstract 2

HDAC2 inhibition prevents endothelial cells from undergoing mesenchymal cell differentiation and reduces the development of cerebral arteriovenous malformations in mouse models.

## Introduction

Cerebral arteriovenous malformations (AVMs) are the most common vascular malformations (1, 2); these consist of arteriovenous (A-V) shunts, which carry blood directly from the arteries to the veins, bypassing brain tissue, that have become abnormal (1–4). (1–4). Due to the vascular abnormalities and elevated blood pressure in the A-V shunts, vessels may ultimately rupture and cause hemorrhagic strokes associated with high mortality (1–5). Of patients who survive, many suffer permanent disability, crippling neurological deficits, seizures, and headaches (1–4). Currently, there are no primary preventative measures for cerebral AVMs (2).

Bone morphogenetic protein (BMP) signaling is involved in cerebral AVMs. Mutations of the BMP type I receptor activin receptor-like kinase 1 (ALK1) result in hereditary hemorrhagic telangiectasia type 2 (HHT2), which is characterized by the presence of AVMs in multiple organs (6, 7). Mutations of endoglin, an ALK1 coreceptor, result in HHT1 (8, 9). Matrix Gla protein (MGP), a BMP inhibitor, is induced by ALK1 signaling and provides feedback regulation for BMP activity (10–14). Loss of MGP causes AVMs in brain, lungs, kidneys, and retina (13, 15, 16). ALK1-deficient mice resemble MGP-deficient mice in that both have reduced MGP (13–15). Importantly, excess MGP limits AVMs in the ALK1-deficient mice, suggesting that lack of MGP may contribute to AVMs in HHT (14). Mutations in the human *Mgp* gene cause an extremely rare disease, referred to as Keutel syndrome, that is associated with severe cardiovascular defects (17–19). However, full evaluations of potential AVMs have not been reported for these rare patients.

Endothelial differentiation is essential for maintaining vascular integrity and homeostasis (16, 20–26). Disturbances in cell signaling can misdirect endothelial differentiation and cause disease (16, 25, 26). In recent years, advanced studies have shown that unwanted cell signaling shifts endothelial differentiation in various vascular diseases. Endothelial lineage cells gain plasticity and acquire other lineage characteristics to become direct pathogenic contributors (25, 27, 28). In this study, we take advantage of recent animal models and suggest that the induction of endothelial histone deacetylase 2 (HDAC2) shifts the transcriptional landscape of cerebral endothelial cells (ECs) toward ill-fated differentiation, leading to cerebral AVMs. We show that limiting HDAC2 prevents this cell shift and reduces the incidence of cerebral AVMs.

## Results

### Cerebral AVMs occur after endothelial-specific deletion of Mgp.

**Figure 1:**
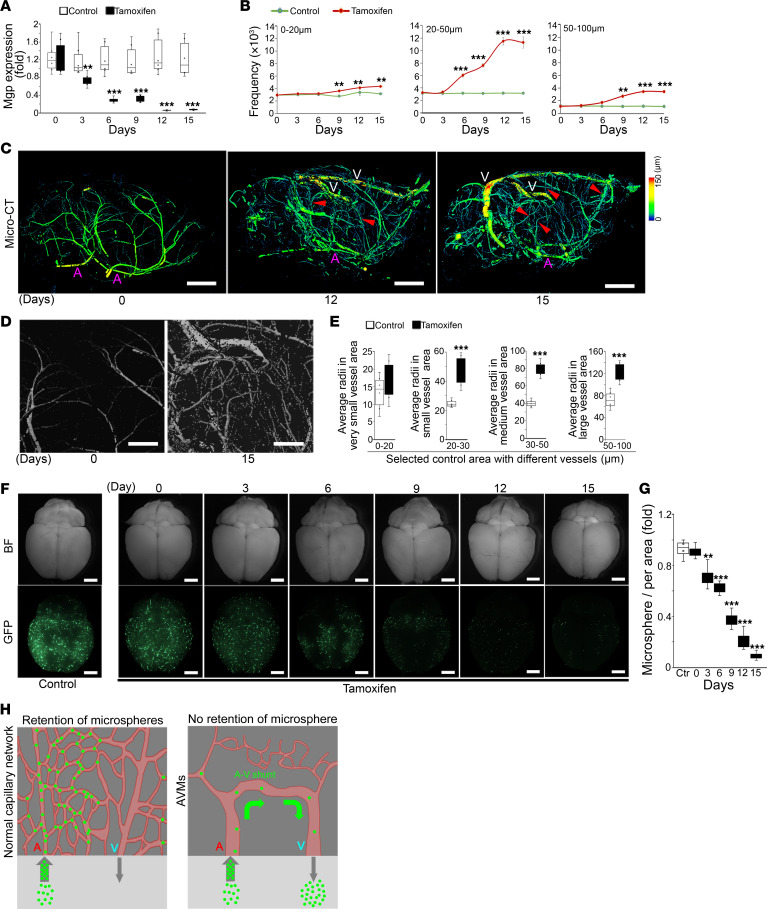
Endothelial-specific deletion of Mgp causes cerebral AVMs. (**A**) Time-course expression of Mgp in CD31^+^CD45^–^ cerebral ECs isolated from *VE-cadherin^cre/ERT2^Mgp^fl/fl^* mice treated with or without tamoxifen (*n* = 6). (**B**) Time course for frequency of brain vessels with different radii in *VE-cadherin^cre/ERT2^Mgp^fl/fl^* mice treated with or without tamoxifen, as determined by micro-CT imaging (*n* = 6). (**C**) Micro-CT imaging of brain vasculature in *VE-cadherin^cre/ERT2^Mgp^fl/fl^* mice on days 12 and 15 after treatment with tamoxifen. Day 0 represents the control treatment. Arrowheads represent A-V shunts. A, artery. V, vein. Scale bars: 1 mm. (**D**) Highlight of A-V shunts on day 15 after treatment with tamoxifen compared with control. Scale bars: 0.5 mm. (**E**) Average radii of vessels in corresponding areas in the control group and tamoxifen-injected group (*n* = 6). (**F** and **G**) A-V shunting as demonstrated by fluorescent microsphere passage with quantitation of retained fluorescent microspheres in the brains of *VE-cadherin^cre/ERT2^Mgp^fl/fl^* mice from day 0 to 15 after treatment with tamoxifen (*n* = 4). BF, bright field. Scale bars: 1 mm. (**H**) Schematic diagram of fluorescent microsphere passage. Data represented in **A**, **B**, and **G** were analyzed for statistical significance by ANOVA with post hoc Tukey’s test. Data represented in **E** were analyzed for statistical significance by unpaired, 2-tailed Student’s *t* test. The bounds of the boxes show upper and lower quartiles with data points. The lines in the boxes show the medians. Error bars represent maximal and minimal values. **P < 0.001; ***P < 0.0001.

To examine the effect of endothelial-specific gene deletion of *Mgp* on cerebral vasculature, we bred *VE-cadherin^cre/ERT2^* mice with recently generated *Mgp^fl/fl^* mice (29). At 10 weeks of age, we administered tamoxifen to the *VE-cadherin^cre/ERT2^Mgp^fl/fl^* mice by intraperitoneal injection for 5 consecutive days. After injection, we examined the time-course expression of MGP in CD31^+^CD45^–^ cerebral ECs isolated from *VE-cadherin^cre/ERT2^Mgp^fl/fl^* mice between day 0 and day 15. Real-time PCR showed that Mgp expression began to decrease on day 3 after tamoxifen administration and continued to decrease to levels of less than 10% on day 12 compared with control treatment (Figure 1A), confirming efficient Mgp deletion in cerebral ECs. We examined the brain vessels using micro-CT imaging and quantitated the frequency of vessels with radii between 10 and 100 μm. The results showed an increase in the frequency of vessels in this range after day 9 (Figure 1B). Specifically, the frequency of vessels with 20–50 μm radii was dramatically increased (Figure 1B), which suggested a disordered lumen, a characteristic of AVMs (30). Micro-CT imaging on days 12 and 15 confirmed the abnormal and enlarged vessels with A-V shunts in the cerebrum of *VE-cadherin^cre/ERT2^Mgp^fl/fl^* mice (Figure 1, C and D). We used *VE-cadherin^cre/ERT2^Mgp^fl/fl^* mice without tamoxifen injection as controls; in these, no abnormalities of brain vasculature were detected by micro-CT when compared with WT mice or *VE-cadherin^cre/ERT2^* mice with tamoxifen injection (Figure 1C and Supplemental Figure 1; supplemental material available online with this article; https://doi.org/10.1172/JCI176758DS1), supporting that loss of MGP was the direct cause of enlarged vessels and A-V shunts.

To determine which vessels were dilated, we quantified the vessel radii in corresponding areas in the control group and the tamoxifen-injected group. We selected areas that contained very small vessels (0–20 μm), small vessels (20–30 μm), medium vessels (30–50 μm), and large vessels (50–100 μm). The results showed that there was a 2-fold increase in 20–30 μm small vessels and 30–50 μm medium vessels and a 1.5-fold increase in 50–100 μm large vessels in the tamoxifen-injected group compared with controls (Figure 1E). We injected the mice with 15 μm fluorescent microspheres to detect A-V shunts (30). The microspheres were retained in the capillaries in the control mice (Figure 1F). Since lack of MGP dramatically increased the vascular lumen size (Figure 1B) and created A-V shunts (Figure 1, C and D), the microspheres were directly washed out through the A-V shunts. Indeed, the imaging showed a decrease in fluorescent density in the cerebrum of *VE-cadherin^cre/ERT2^Mgp^fl/fl^* mice on day 3 after tamoxifen administration and a further decrease from days 3 to 15 (Figure 1, F and G), supporting the appearance of enlarged lumens and A-V shunts (Figure 1H). Together, the results suggest that endothelial-specific gene deletion of *Mgp* caused cerebral AVMs in mice.

### Cerebral ECs acquire mesenchymal markers after Mgp deletion and cause vascular malformation.

**Figure 2:**
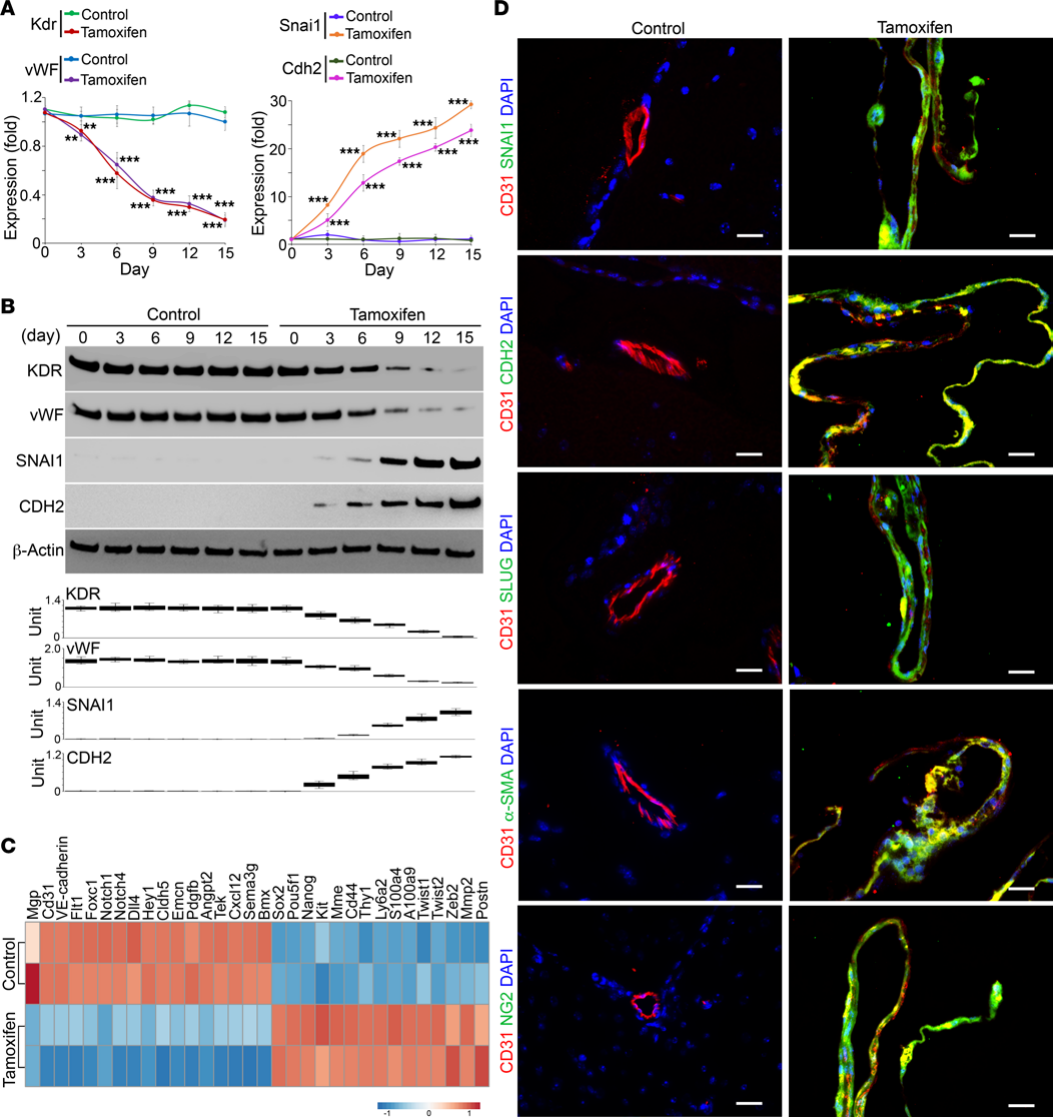
Mgp-deleted cerebral ECs acquire mesenchymal markers. (**A** and **B**) Time-course expression of endothelial and mesenchymal markers in CD31^+^CD45^–^ cerebral ECs isolated from *VE-cadherin^cre/ERT2^Mgp^fl/fl^* mice treated with or without tamoxifen, shown by real-time PCR (**A**) and immunoblotting with densitometry (**B**) (*n* = 6). (**C**) Heatmap of the RNA-Seq of CD31^+^CD45^–^ cerebral ECs isolated from *VE-cadherin^cre/ERT2^Mgp^fl/fl^* mice on day 9 after treatment with or without tamoxifen. Each row of the heatmap represents an independent group in which the cells were isolated and pooled from 3 mice. Marker genes were differentially expressed, with statistical significance set at an adjusted *P* value of less than 0.05. (**D**) Coimmunostaining of endothelial marker CD31 and mesenchymal markers in the cerebrums of *VE-cadherin^cre/ERT2^Mgp^fl/fl^* mice on day 15 after treatment with or without tamoxifen (*n* = 6). Scale bars: 20 μm. Data represented in **A** were analyzed for statistical significance by ANOVA with post hoc Tukey’s test. The bounds of the boxes show upper and lower quartiles with data points. The lines in the boxes show the medians. Error bars represent maximal and minimal values. **P < 0.001; ***P < 0.0001.

To investigate endothelial differentiation in the cerebral AVMs, we again isolated CD31^+^CD45^–^ cerebral ECs from *VE-cadherin^cre/ERT2^Mgp^fl/fl^* mice at different time points after tamoxifen injection. Real-time PCR and immunoblotting showed a gradual decrease in the expression of Kdr and vWF together with an increase in the expression of Snai1 and Cdh2 (Figure 2, A and B). The results suggested that the cerebral ECs progressively acquired mesenchymal cell markers. We performed RNA-Seq using CD31^+^CD45^–^ cerebral ECs on day 9 and found that additional mesenchymal and stem cell markers (25, 31) were induced with the decrease in endothelial markers (Figure 2C). We also costained CD31 with mesenchymal markers in *Mgp^–/–^* cerebrums on day 15 after tamoxifen injection. We found that more mesenchymal markers, such as SLUG, α-smooth muscle actin (α-SMA), and neuron-glial antigen 2 (NG2), appeared in the CD31^+^ ECs in the vascular malformation (Figure 2D). The results supported that an ongoing mesenchymal transition occurred in cerebral ECs when the cells were losing MGP, causing the cells to acquire mesenchymal characteristics.

**Figure 3:**
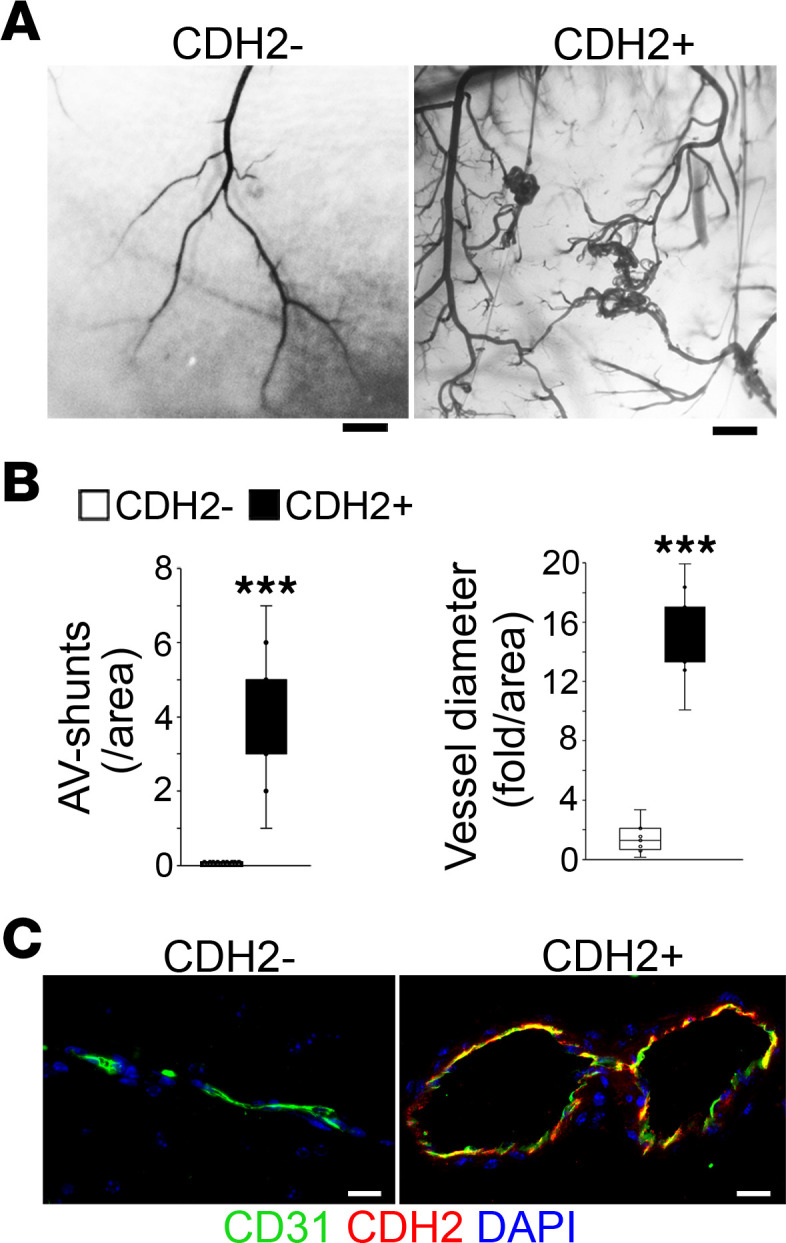
Mgp-deleted cerebral ECs acquire mesenchymal markers and cause vascular malformations during vascular repair. (**A**) Latex dye staining with tissue clearing at the repairing sites after EC transplantation (*n* = 9). Scale bars: 300 μm. (**B**) Quantification of A-V shunts and vessel diameters after tissue clearing at the repair sites after EC transplantation (*n* = 6). (**C**) Coimmunostaining of CD31, CDH2, and DAPI after tissue clearing at the repair sites after EC transplantation. Scale bars: 50 μm. Data represented in **B** were analyzed for statistical significance by unpaired, 2-tailed Student’s *t* test. The bounds of the boxes show upper and lower quartiles with data points. The lines in the boxes show the medians. Error bars represent maximal and minimal values. ***P < 0.0001.

To determine whether the cerebral ECs with mesenchymal cell markers cause vascular malformation, we transplanted the cells in a model of hind-limb ischemia in nude mice. Since revascularization occurred in the ischemic area after the procedure, this model of hind-limb ischemia has been used to evaluate the capacity for vascular repair of implanted ECs (32). We adopted this method to determine whether implanting cerebral ECs that acquired mesenchymal characteristics caused vascular malformation during revascularization. On day 15 after tamoxifen injection, we isolated a CD31^+^CD45^–^ subpopulation that coexpressed CDH2 (CDH2^+^) and transplanted the cells into nude mice, in which the proximal and distal femoral arteries were ligated. Cells without CDH2 expression (CDH2^–^) were used as controls. Two weeks after transplantation, latex dye staining with tissue clearing showed enlarged and twisted small vessels at the repair sites in mice transplanted with CDH2^+^ cells (Figure 3, A and B). We costained the vessels with anti-CD31 and anti-CDH2 antibodies. The results showed colocalization of CD31 and CDH2 in the vessels where CDH2^+^ cells had been transplanted (Figure 3C), supporting that cerebral ECs undergoing mesenchymal cell differentiation caused AVMs.

### Limiting HDAC2 prevents mesenchymal differentiation in cerebral ECs and reduces cerebral AVMs.

**Figure 4:**
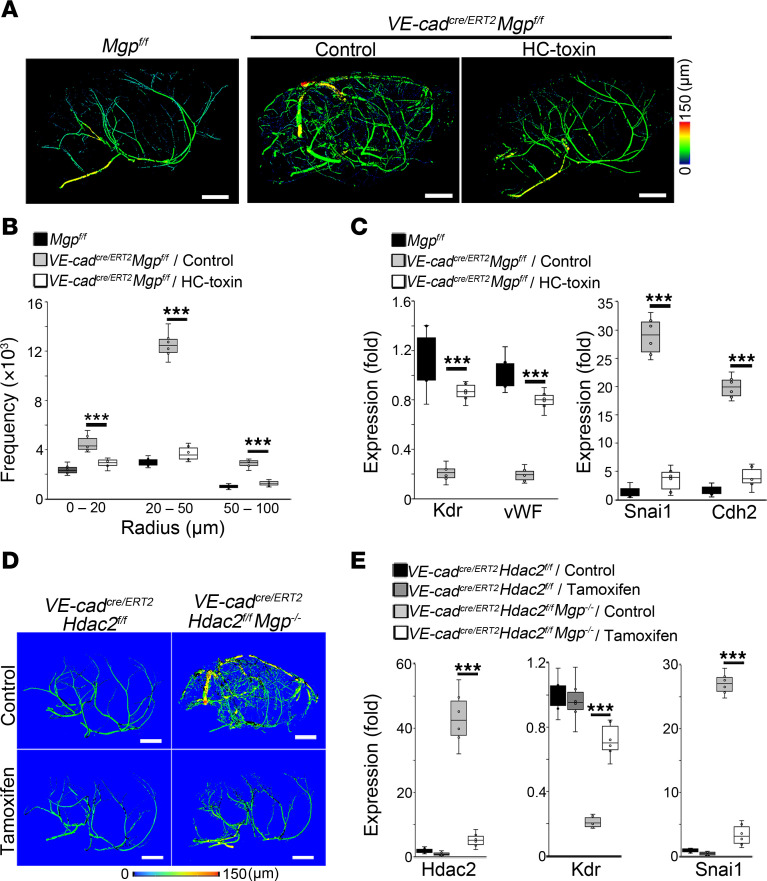
Limiting HDAC2 prevents cerebral ECs from undergoing mesenchymal differentiation and reduces cerebral AVMs. (**A**) Micro-CT imaging of brain vasculature in *VE-cadherin^cre/ERT2^Mgp^fl/fl^* mice treated with tamoxifen in combination of HC toxin (*n* = 6). Scale bar: 1 mm. (**B**) Frequency of vessels with different radii in the cerebrums of *VE-cadherin^cre/ERT2^Mgp^fl/fl^* mice treated with tamoxifen in combination with HC toxin as determined by micro-CT imaging (*n* = 6). (**C**) Expression of endothelial and mesenchymal markers in CD31^+^CD45^–^ cerebral ECs isolated from *VE-cadherin^cre/ERT2^Mgp^fl/fl^* mice after treatment with a combination of tamoxifen and HC toxin, as determined by real-time PCR (*n* = 6). (**D**) Micro-CT imaging of brain vasculature in *VE-cadherin^cre/ERT2^Hdac2^fl/fl^Mgp^–/–^* mice and *VE-cadherin^cre/ERT2^Hdac2^fl/fl^* mice treated with tamoxifen (*n* = 6). Scale bars: 1 mm. (**E**) Expression of endothelial and mesenchymal markers in CD31^+^CD45^–^ cerebral ECs isolated from *VE-cadherin^cre/ERT2^Hdac2^fl/fl^Mgp^–/–^* mice and *VE-cadherin^cre/ERT2^Hdac2^fl/fl^* mice treated with tamoxifen, as determined by real-time PCR (*n* = 6). Data represented in **B**, **C**, and **E** were analyzed for statistical significance by ANOVA with post hoc Tukey’s test. The bounds of the boxes show upper and lower quartiles with data points. The lines in the boxes show the medians. Error bars represent maximal and minimal values. ***P < 0.0001.

To search for compounds that would reduce cerebral AVMs, we used an established cell model, MGP CRISPR cells, in which MGP was depleted in human brain microvascular ECs (HBMECs) by CRISPR/Cas9 gene editing (30, 33, 34). MGP CRISPR cells showed mesenchymal characteristics with Snai1 expression and recapitulated the findings for the ECs in cerebral AVMs (30). We integrated Snai1 promoter–driven GFP into MGP CRISPR cells to generate a cell line with high GFP intensity (Supplemental Figure 2). We screened compound libraries and found that HC toxin, a HDAC2 inhibitor, reduced the intensity of GFP (Supplemental Figure 2), suggesting that HC toxin might reduce mesenchymal characteristics in brain ECs. We treated *VE-cadherin^cre/ERT2^Mgp^fl/fl^* mice at 10 weeks of age with a combination of tamoxifen and HC toxin (5 μg/kg, daily) for 5 days and continued HC-toxin treatment until 2 weeks. The HC-toxin treatment was initiated before AVM formation. After the treatment, micro-CT imaging showed a dramatic improvement of the cerebral vasculature, with a reduction of AVMs in the HC toxin–treated group compared with saline-treated controls (Figure 4A). Radius analysis further revealed that the frequency of vessels with radii between 10 and 100 μm in the HC toxin–treated group had normalized when compared with that of controls (Figure 4B). The results suggested that the HDAC2 inhibitor reduced cerebral AVMs in *VE-cadherin^cre/ERT2^Mgp^fl/fl^* mice. We then isolated CD31^+^CD45^–^ cerebral ECs from the HC toxin–treated group and controls. Real-time PCR showed that HC toxin prevented the decrease in expression of the endothelial markers Kdr and vWF and abolished the induction of the mesenchymal markers Snai1 and Cdh2 (Figure 4C), suggesting that HDAC2 inhibition rescued cerebral ECs from mesenchymal differentiation.

To determine whether endothelial-specific deletion of Hdac2 affected cerebral AVMs, we bred *VE-cadherin^cre/ERT2^Hdac2^fl/fl^* mice with *Mgp^–/–^* mice, in which cerebral AVMs occurred at 4–6 weeks of age (16, 30). We treated *VE-cadherin^cre/ERT2^Hdac2^fl/fl^Mgp^–/–^* mice at 1 week of age, prior to the formation of AVMs, with tamoxifen for 5 days to delete endothelial Hdac2. At 4 weeks of age, micro-CT imaging showed a robust difference in the cerebral vasculature between the group injected with tamoxifen and untreated controls (Figure 4D). Without tamoxifen administration, abnormal networks of cerebral vessels with A-V shunts were detected in the *VE-cadherin^cre/ERT2^Hdac2^fl/fl^Mgp^–/–^* mice (Figure 4D). With tamoxifen administration, the cerebral vasculature normalized, with a reduction in AVMs (Figure 4D). The CD31^+^CD45^–^ cerebral ECs were isolated, and real-time PCR results revealed that gene deletion of *Hdac2* blocked the reduction of endothelial markers and prevented the induction of mesenchymal markers (Figure 4E). Together, the results support that limiting endothelial HDAC2 rescued cerebral ECs from mesenchymal differentiation and reduced cerebral AVMs.

### Alterations in expression of HDAC2 and EZH1 in cerebral ECs after MGP deletion.

**Figure 5:**
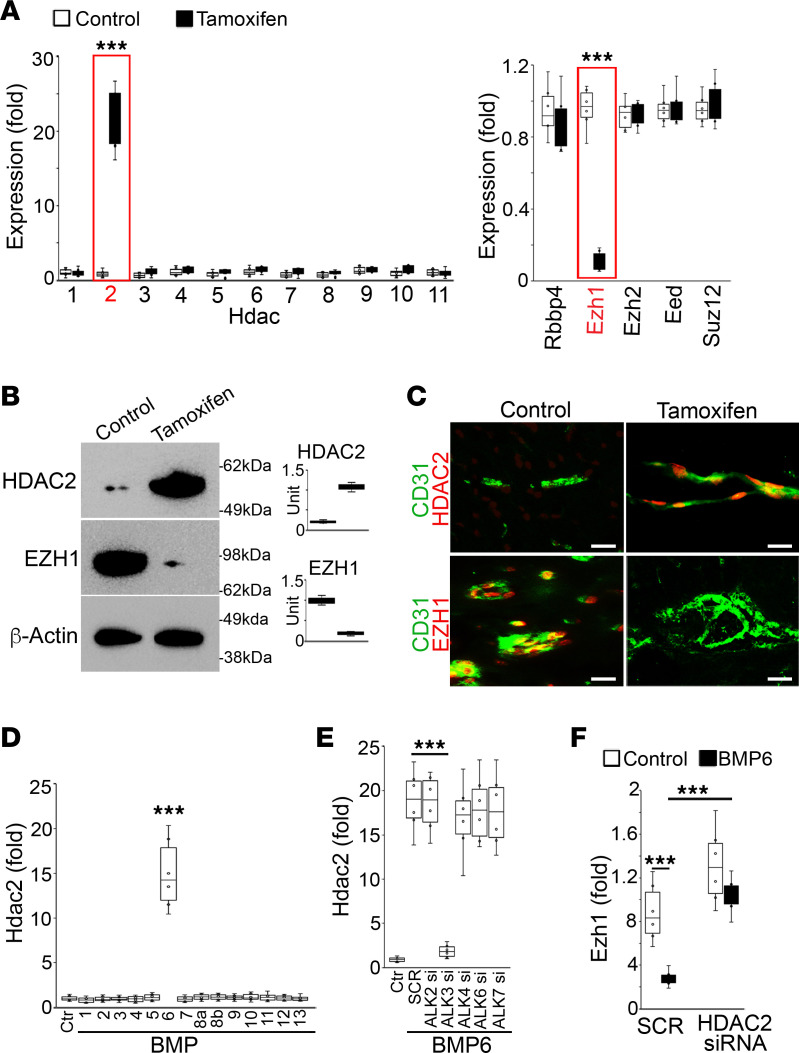
Alterations in the expression of HDAC2 and EZH1 in cerebral ECs after Mgp deletion. (**A**) Expression of Hdac1 to -11, Ezh1, Rbbp4, Ezh2, Eed, and Suz12 in CD31^+^CD45^–^ cerebral ECs isolated from *VE-cadherin^cre/ERT2^Mgp^fl/fl^* mice treated with or without tamoxifen, as determined by real-time PCR (*n* = 6). (**B**) Expression of HDAC2 and EZH1 in CD31^+^CD45^–^ cerebral ECs isolated from *VE-cadherin^cre/ERT2^Mgp^fl/fl^* mice treated with or without tamoxifen, as determined by immunoblotting. β-Actin was used as loading control. (**C**) Coimmunostaining of HDAC2 and EZH1 with CD31 in the cerebral vasculature of *VE-cadherin^cre/ERT2^Mgp^fl/fl^* mice treated with or without tamoxifen (*n* = 8). Scale bars: 50μm. (**D**) BMP-6–induced Hdac2 in HBMECs as shown by real-time PCR (*n* = 6). (**E**) Depletion of ALK-3 abolished the induction of Hdac2 in BMP-6–treated HBMECs (*n* = 6). (**F**) BMP-6 reduced Ezh1, and Hdac2 depletion restored Ezh1 expression (*n* = 6). Data represented in **A** and **F** were analyzed for statistical significance using unpaired, 2-tailed Student’s *t* test. Data represented in **D** and **E** were analyzed for statistical significance using ANOVA with post hoc Tukey’s test. The bounds of the boxes show upper and lower quartiles with data points. The lines in the boxes show the medians. ***P < 0.0001.

To determine the role of HDAC2 in cerebral AVMs, we first examined HDAC2 expression in MGP-deleted cerebral ECs. CD31^+^CD45^–^ cerebral ECs were isolated from *VE-cadherin^cre/ERT2^Mgp^fl/fl^* mice at 2 weeks after tamoxifen administration. Nontamoxifen administration was used as control. The expression of members of the HDAC family, including HDAC1 to -11, was examined. Real-time PCR showed that only Hdac2 was induced in the MGP-deleted cerebral ECs (Figure 5A). Interestingly, when we examined the components of the polycomb repressive complex that mediate histone methylation, including enhancer of zeste homolog 1 (Ezh1), Ezh2, suppressor of zeste 12 (Suz12), embryonic ectoderm development protein (Eed), and retinoblastoma-binding protein 4 (Rbbp4) (35, 36), we found a decrease in Ezh1 in the cerebral ECs (Figure 5A). Immunoblotting confirmed the induction of HDAC2 with the reduction of EZH1 in MGP-deleted cerebral ECs (Figure 5B). Similarly, immunostaining revealed the increased HDAC2 and the decreased EZH1 in CD31^+^ cerebral ECs of enlarged vessels (Figure 5C). The results suggest that alterations in HDAC2 and EZH1 are involved in cerebral AVM formation after MGP deletion.

Since MGP functions as a BMP inhibitor (10), we treated HBMECs with different BMP ligands and examined Hdac2 expression. Real-time PCR showed that only BMP-6 was able to induce Hdac2 (Figure 5D). To determine which BMP type I receptor mediated Hdac2 induction, we used siRNA individually to deplete the receptors that might interact with BMP-6, including ALK2, -3, -4, -6, and -7. The results showed that deletion of ALK-3 prevented BMP-6 from inducing Hdac2 (Figure 5E), suggesting that loss of MGP enhanced activity of the BMP-6/ALK3 pathway to induce Hdac2. We also examined the expression of Ezh1 in HBMECs after BMP-6 treatment and found a reduction of Ezh1 (Figure 5F); the level of Ezh1 was rescued by a depletion of Hdac2 using siRNA. The results suggested that loss of MGP elevated the activity of BMP-6/ALK3 to induce Hdac2 and in turn suppress Ezh1.

### Alterations in expression of HDAC2 and EZH1 in human cerebral AVMs correlate with the induction of mesenchymal marker and lumen enlargement.

**Figure 6:**
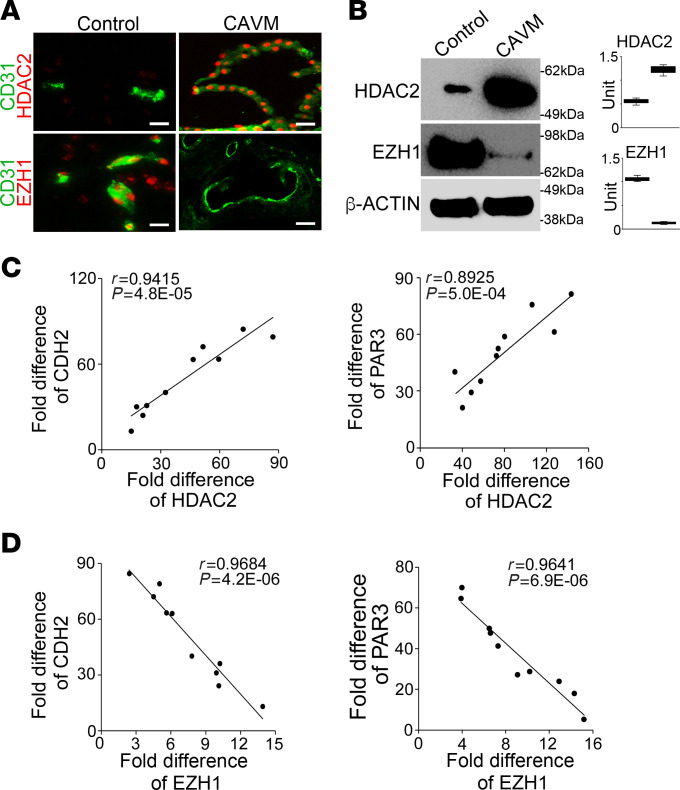
Alterations in HDAC2 and EZH1 in human cerebral AVMs correlate with the induction of mesenchymal markers and lumen enlargement. (**A**) Coimmunostaining of HDAC2 and EZH1 with CD31 in human cerebral AVMs (*n* = 5). Scale bars: 50 μm. (**B**) Expression of HDAC2 and EZH1 in human cerebral AVMs, as determined by immunoblotting (*n* = 5). β-Actin was used as loading control. (**C**) Correlation between the fold increase in the expression of the mesenchymal marker CDH2 and the lumen-associated gene PAR3 with HDAC2 in human cerebral AVMs (*n* = 10). (**D**) Correlation between the fold change in the expression of the mesenchymal marker CDH2 and the lumen-associated gene PAR3 with EZH1 in human cerebral AVMs (*n* = 10). Data represented in **C** and **D** were analyzed using Pearson’s correlation coefficient.

To determine whether the alterations in HDAC2 and EZH1 were also present in human cerebral AVMs, we first examined their expression in lesions of human cerebral AVMs. Immunostaining revealed an HDAC2 induction together with an EZH1 reduction in the CD31^+^ cerebral ECs (Figure 6A), which was confirmed by immunoblotting with densitometry (Figure 6B). We further determined the relationship between alterations in HDAC2 and EZH1, endothelial-mesenchymal transitions, and lumen formation in human cerebral AVMs. We performed correlation analysis to assess the expression of HDAC2, EZH1, CDH2, and a lumen-associated gene, PAR3 (32). The results showed strong positive correlations among excess HDAC2, CDH2, and PAR3 (Figure 6C) and negative correlations among expression of EZH1, CDH2, and PAR3 (Figure 6D). The results further supported the possibility that alterations in HDAC2 and EZH1 shift ECs toward mesenchymal differentiation, leading to disruptions in lumen formation in cerebral AVMs.

### Alterations in HDAC2 and EZH1 cause alterations in H4K8ac and H3K27me in the regulation of endothelial and mesenchymal differentiation.

**Figure 7:**
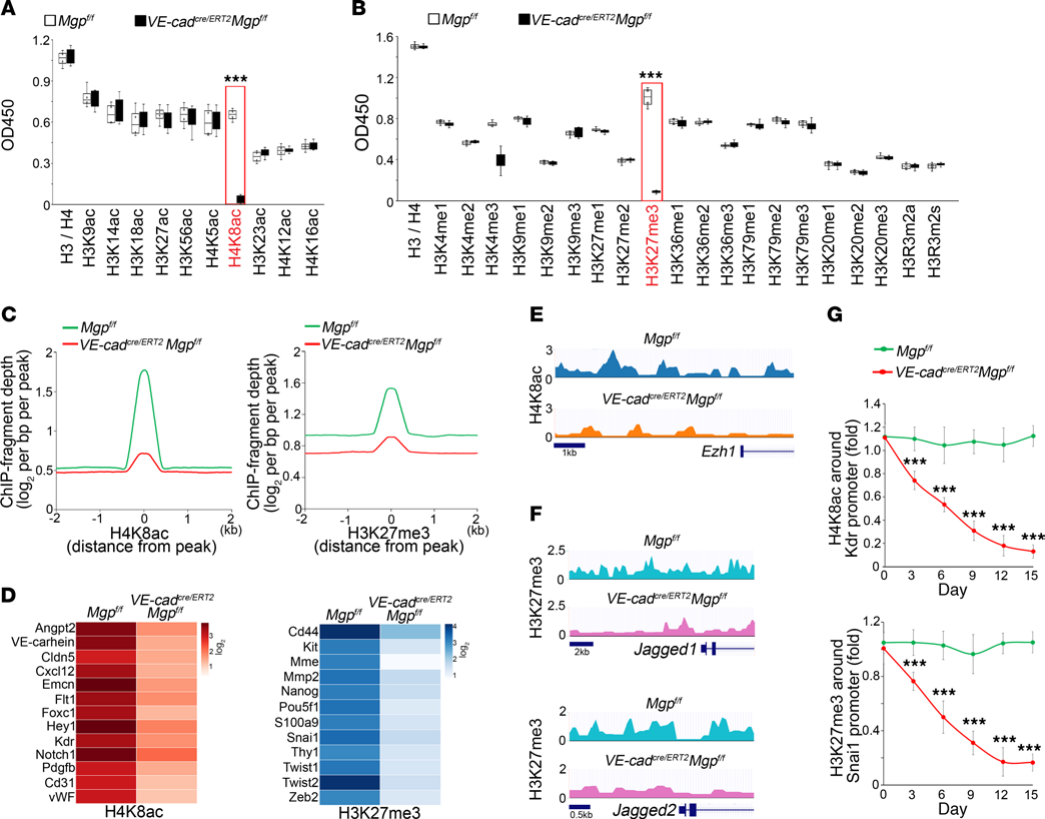
Alterations in H4K8ac and H3K27me3 in gene regulations of endothelial and mesenchymal differentiation. (**A** and **B**) Histone acetylation (**A**) and methylation (**B**) in CD31^+^CD45^–^ cerebral ECs isolated from *VE-cadherin^cre/ERT2^Mgp^fl/fl^* mice treated with tamoxifen. *Mgp^fl/fl^* mice were used as control (*n* = 4). (**C**) ChIP-Seq of H4K8ac and H3K27me3 in CD31^+^CD45^–^ cerebral ECs isolated from *VE-cadherin^cre/ERT2^Mgp^fl/fl^* and *Mgp^fl/fl^* mice treated with tamoxifen. (**D**) Heatmap of the occupancy of H4K8ac around the regulatory regions of endothelial markers and of H3K27me3 around the regulatory regions of mesenchymal and stem cell markers. Each ChIP library was constructed using cells isolated and pooled from 3 mice. Peaks were identified with more than 4-fold enrichment and an FDR of 0.1% compared with controls; log_2_-transformed peak densities were then used to generate occupancy heatmaps. (**E**) Plots of the occupancy of H4K8ac at the Ezh1 gene locus from ChIP-Seq data. (**F**) Plots of the occupancy of H3K27me3 at the Jagged1 and Jagged2 gene loci from ChIP-Seq data. (**G**) Time course of ChIP assays of H4K8ac and H3K27me3 in the promoters of Kdr and Snai1 in CD31^+^CD45^–^ cerebral ECs isolated from *VE-cadherin^cre/ERT2^Mgp^fl/fl^* and *Mgp^fl/fl^* mice treated with tamoxifen (*n* = 6). Data represented in **A** and **B** were analyzed for statistical significance using unpaired, 2-tailed Student’s *t* test. Data represented in **G** were analyzed for statistical significance using ANOVA with post hoc Tukey’s test. The bounds of the boxes show upper and lower quartiles with data points. The lines in the boxes show the medians. Error bars represent maximal and minimal values. ***P < 0.0001.

HDAC2 is an enzyme that specifically removes acetyl groups from ε-*N*-acetyl lysine in histones, whereas EZH1 catalyzes histone methylation (37–40). To determine whether the changes in HDAC2 and EZH1 altered histone modifications, we screened histone acetylation and methylation in CD31^+^CD45^–^ cerebral ECs from *VE-cadherin^cre/ERT2^Mgp^fl/fl^* mice at 2 weeks after tamoxifen injection. We found a distinct decrease of H4K8ac and H3K27me3 (Figure 7, A and B). ChIP-Seq identified 20,606 peaks of H4K8ac in control cerebral ECs and 4,087 peaks of H4K8ac after MGP deletion. The results also showed 41,775 peaks of H3K27me3 in control cerebral ECs and 15,300 peaks of H3K27me3 after MGP deletion. Histogram of ChIP-fragment depth relative to the combined peaks from 2 groups revealed a decrease in coverage of both H4K8ac and H3K27me in the cerebral ECs after MGP deletion (Figure 7C). The results suggest that genome-wide occupancies of both H4K8ac and H3K27me3 were decreased in MGP-depleted cerebral ECs. We further analyzed the ChIP-Seq data and found decreased occupancy of H4K8ac around the regulatory regions of genes involved in endothelial differentiation and decreased occupancy of H3K27me3 around the regulatory regions of genes involved in mesenchymal and stem cell differentiation (Figure 7D). We also showed a decreased occupancy of H4K8ac around the regulatory region of the Ezh1 gene (Figure 7E). The results supported that Hdac2 induction reduced H4K8ac to suppress endothelial differentiation and Ezh1 expression, whereas the decreased EZH1 reduced H3K27me3 to allow mesenchymal and stem cell differentiation in cerebral ECs. In a previous study, we showed an induction of JAGGED 1 and 2 in cerebral AVMs (16). Interestingly, we found a large decrease of H3K27me3 in the regulatory regions of Jagged 1 and 2 in cerebral ECs from the *VE-cadherin^cre/ERT2^Mgp^fl/fl^* mice (Figure 7F), suggesting that a decrease of H3K27me3 caused by altered HDAC2 and EZH1 also resulted in an increase of JAGGED 1 and 2.

We also performed ChIP assays using CD31^+^CD45^–^ cerebral ECs from *VE-cadherin^cre/ERT2^Mgp^fl/fl^* mice at different time points after tamoxifen injection to examine the occupancy of H4K8ac around the Kdr promoter and H3K27me3 around the Snai1 promoter. Previous studies identified the region around –225 to +268 bp in the Kdr promoter and the region around –612 to +90 bp in the Snai1 promoter as critical for the activation of expression (41, 42). Therefore, we examined the occupancy of H4K8ac and H3K27me3 around these regions. The results revealed a progressively decreasing occupancy of H4K8ac around the Kdr promoter, resulting in a suppression of Kdr after MGP deletion (Figure 7G), and of H3K27me3 around the Snai1 promoter, resulting in an activation after MGP deletion (Figure 7G).

**Figure 8:**
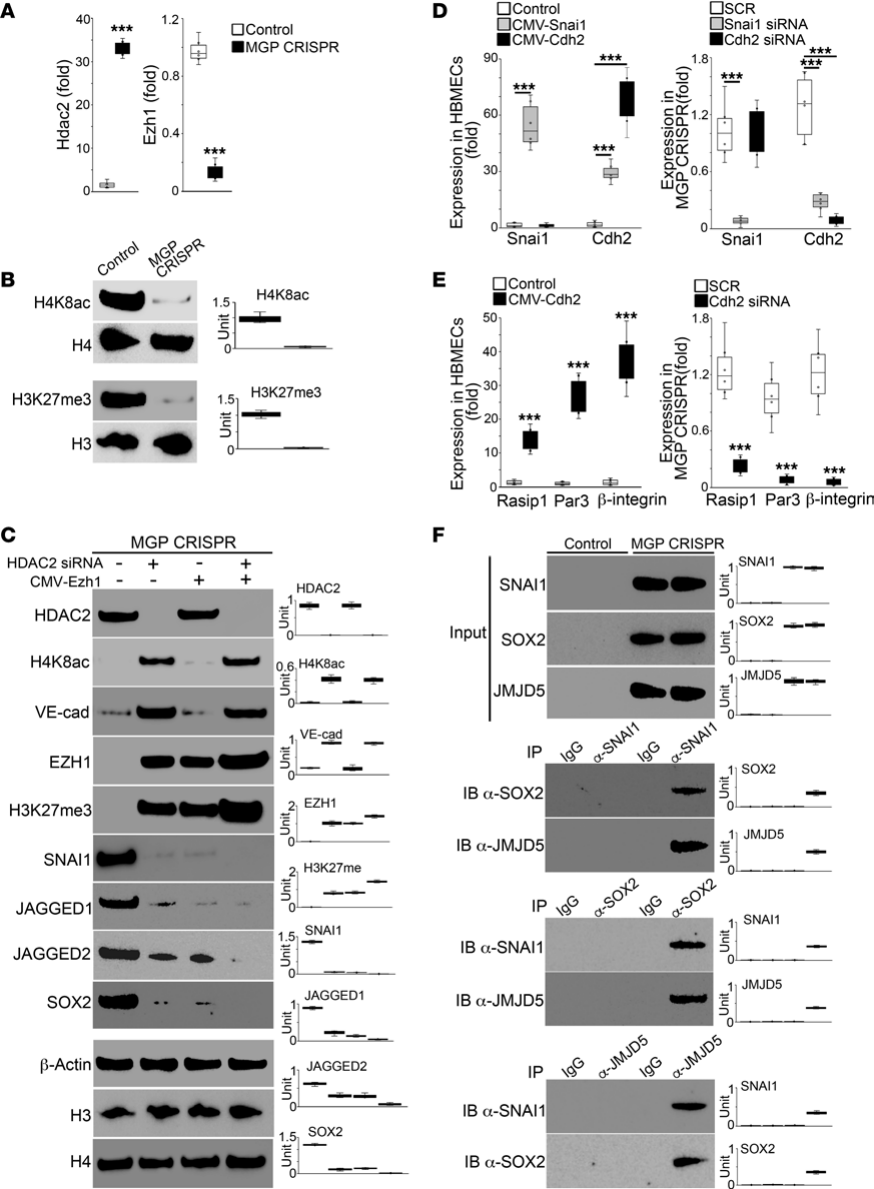
Alterations in HDAC2 and EZH1 cause changes in H4K8ac and H3K27me3 in the regulation of endothelial and mesenchymal differentiation. (**A**) Expression of Hdac2 and Ezh1 in MGP CRISPR cells and control HBMECs shown by real-time PCR (*n* = 6). (**B**) Abundance of H4K8ac and H3K27me3 in MGP CRISPR cells and control HBMECs shown by immunoblotting with densitometry. H3 and H4 were used as loading controls. (**C**) Expression of HDAC2, VE-cadherin, SNAI1, EZH1, JAGGED1, JAGGED2, and SOX2 and abundance of H4K8ac and H3K27me3 in MGP CRISPR cells after transfection with HDAC2 siRNA or infection with CMV-Ezh1 lentivirus or a combination of both, as shown by immunoblotting with densitometry. CMV, cytomegalovirus promoter. β-Actin, H3, and H4 were used as loading controls. (**D**) Expression of Snai1 and Cdh2 in HBMECs or MGP CRISPR cells after overexpression or depletion of Snai1 and Cdh2 (*n* = 6). (**E**) Expression of lumen-associated genes in HBMECs or MGP CRISPR cells after overexpression or depletion of Cdh2 (*n* = 6). (**F**) Immunoblotting with densitometry after coimmunoprecipitation with anti-SNAI1, anti-SOX2, or anti-JMJD5 antibodies in HBMECs or MGP CRISPR cells. Data represented in **A** and **E** were analyzed for statistical significance using unpaired, 2-tailed Student’s *t* test. Data represented in **D** were analyzed for statistical significance using ANOVA with post hoc Tukey’s test. The bounds of the boxes show upper and lower quartiles with data points. The lines in the boxes show the medians. Error bars represent maximal and minimal values. ***P < 0.0001.

To further explore the role of HDAC2 and EZH1 in modifying H4K8ac and H3K27me3 to shift the EC differentiation, we used MGP CRISPR cells. Real-time PCR showed robust induction of Hdac2 as well as reduction of Ezh1 in MGP CRISPR cells (Figure 8A), and immunoblotting revealed decreases in H4K8ac and H3K27me3 (Figure 8B). We then depleted HDAC2 in the MGP CRISPR cells using Hdac2 siRNA. The immunoblotting showed restored levels of H4K8ac, EZH1, and H3K27me3. Depletion of HDAC2 also normalized the EC markers and abolished the mesenchymal markers (Figure 8C). We overexpressed EZH1 in MGP CRISPR cells using lentiviral vectors containing CMV promoter–driven Ezh1 cDNA. The results showed that the excess EZH1 only restored H3K27me3 and abolished mesenchymal markers, but had no effect on EC markers (Figure 8C). When HDAC2 depletion and excess EZH1 were combined, immunoblotting showed enhanced H3K27me3 levels and further reduction of mesenchymal markers without affecting H4K8ac and EC markers (Figure 8C). In our previous study, we found that JAGGED 1 and 2 induced SOX2 in cerebral AVMs (33). Interestingly, ChIP-Seq in this study showed that altered HDAC2 and EZH1 caused a decrease in H3K27me3 in the regulatory regions of Jagged 1 and 2 (Figure 7F). To determine whether altered HDAC2 and EZH1 elevated JAGGED 1 and 2 after MGP deletion for SOX2 induction, we examined the expression of JAGGED 1 and 2 and SOX2 in MGP CRISPR cells after depletion of HDAC2 or overexpression of EZH1. The results showed that either HDAC2 depletion or excess EZH1 reduced the expression of JAGGED 1 and 2 and SOX2 (Figure 8C). The results suggested that lack of MGP caused induction of HDAC2, which led to a reduction of EZH1 that decreased H3K27me and allowed an elevation of JAGGED 1 and 2 to induce SOX2.

Lumen disorder, caused by dysregulation of lumen-associated genes, is a main characteristic of human cerebral AVMs (30). To determine the relationship between expression of Snai1 or Cdh2 and lumen-associated genes, we overexpressed Snai1 or Cdh2 in HBMECs. Real-time PCR showed that the overexpression of Snai1 induced Cdh2, but overexpression of Cdh2 did not affect Snai1 expression (Figure 8D). We depleted Snai1 or Cdh2 in MGP CRISPR cells, which showed that depletion of Snai1 abolished the expression of Cdh2, but again, that depletion of Cdh2 did not affect Snai1 expression (Figure 8D). The results suggested that elevated Snai1 induced Cdh2 after deletion of MGP. To determine whether Cdh2 was involved in the AVM formation, we overexpressed Cdh2 in HBMECs or depleted it in MGP CRISPR cells and examined lumen-associated genes, such as Rasip1, Par3, and β-integrin, which are associated with cerebral AVMs (30). Real-time PCR showed that overexpression of Cdh2 induced these lumen-associated genes in HBMECs whereas depletion of Cdh2 reduced the expression of these genes in MGP CRISPR cells (Figure 8E). The results suggested that Snai1-induced Cdh2 was involved in the disordered lumen after MGP deletion.

We previously showed that SOX2 elevated jumonji domain–containing protein 5 (JMJD5) and formed a complex to induce Cdh2 (30). In this study, we showed that Snai1 induced Cdh2 to alter lumen-associated genes (Figure 8, D and E). To determine whether SNAI1 interacted with the complex formed by SOX2 and JMJD5 for Cdh2 induction, we performed coimmunoprecipitation using MGP CRISPR cells. The immunoblotting showed that SNAI1 interacted with SOX2 and JMJD5 (Figure 8F), suggesting that loss of MGP caused an induction of SNAI1, SOX2, and JMJD5 to form a complex that induced Cdh2 and caused lumen disorder.

Together, the results suggested that ongoing alterations in the H4K8ac and H3K27me3 histone modifications caused by HDAC2 induction unremittingly shifted the transcriptional landscape of ECs toward mesenchymal cell differentiation in cerebral AVMs.

## Discussion

In this study, we used the recently generated *Mgp^fl/fl^* mice to create a model for studying endothelial differentiation in cerebral AVMs. We found severe cerebral AVMs in the *Mgp^fl/fl^* mice after endothelial-specific MGP deletion. Using this model, we examined cerebral EC differentiation and discovered that cerebral ECs acquired mesenchymal markers after MGP deletion. We found that these ill-fated ECs formed A-V shunts during vascular repair. In both human cerebral AVMs and MGP-null cerebral ECs, there was a striking HDAC2 induction together with a reduction of EZH1. Our results suggested that HDAC2 induction specifically decreased H4K8ac to limit cerebral EC differentiation and reduce EZH1 expression. Decreased EZH1 diminished H3K27me3 and unleashed mesenchymal cell differentiation in the ECs. We showed that an inhibitor of HDAC2 or endothelial-specific deletion of HDAC2 prevented the ECs from undergoing mesenchymal cell differentiation, which substantially reduced the development of cerebral AVMs.

The HDAC family is a group of enzymes that specifically remove acetyl groups from ε-*N*-acetyl lysines of histones to change chromatin structure and control transcription (37). Mammals carry 11 canonical HDACs, which show variable activity in different tissues (43). Previous studies have clarified some aspects of endothelial HDACs. HDAC1 and -3 mediate the cellular response to oscillatory shear stress in ECs (44). Lack of HDAC7 damages EC–cell adhesion (45). HDAC4, -5, and -7 regulate VEGF activity and affect angiogenesis (46–48). However, the function of HDAC2 in cerebral ECs has been unclear. Here, we find that the induction of HDAC2 alters specific histone modifications in ECs to shift their fate and lead to cerebral AVMs. The study provides insights in the role of epigenetic regulation in cerebral AVMs.

Previous studies have suggested that histone modifications are important in maintaining the integrity of ECs and that impaired histone modifications affect vascular development (49–54). H4K8ac is the acetylation at the 8th lysine in histone H4, which is abundant in promoters that are transcriptionally active (55). H3K27me3 is the trimethylation on the 27th lysine of histone H3 that suppresses nearby genes (56). The activity of H3K27me3 is mediated by a polycomb repressive complex including EZH1, EZH2, SUZ12, EED, and RBBP4 (35, 36). In particular, EZH1 catalyzes H3K27me3 to suppress undesired differentiation in stem cells (38–40). In this study, our results suggest that induction of HDAC2 reduces EZH1 expression, which diminishes H3K27me3 and induces mesenchymal differentiation in the ECs. When combined with interrupted EC differentiation due to the decrease of H4K8ac, this decreased EZH1 will drive ECs toward mesenchymal differentiation in cerebral AVMs.

Transcriptomic data of brain cells from a publicly available source (57) show that MGP is strongly expressed in brain pericytes and moderately expressed in brain ECs (Supplemental Figure 3). However, MGP activity is known to affect extracellular matrix and microenvironment very locally (58). We have reported that lack of MGP in pulmonary ECs, where MGP is expressed at low levels, still caused ECs to undergo unwanted differentiation to contribute myofibroblasts to pulmonary fibrosis (29). This study is another example of the important role of MGP in EC differentiation regardless of expression level. However, it would be interesting to investigate whether cerebral AVMs worsen after deletion of MGP in both brain pericytes and ECs.

## Methods

### Sex as a biological variable

Mixed groups of male and female mice were used in this study.

### Animals

The *VE-cadherin^cre/ERT2^* mice were obtained as gifts from Ralf Adams (Max Planck Institute for Molecular Biomedicine, Münster, Germany), and the *Mgp^fl/fl^* mouse was from our breeding colony (29, 59). Hdac2*^fl/fl^* (B6.Cg-Hdac2tm1.1Rdp/J) and *Mgp^+/–^ (B6.129S7-Mgptm1Kry/KbosJ)* mice were obtained from the Jackson Laboratory. Genotypes were confirmed by PCR (11), and experiments were performed with generation F4–F6. Littermates were used as controls. All mice were fed a standard chow diet (diet 8604, Harlan Teklad Laboratory). Tamoxifen (Sigma-Aldrich, T5648) was injected for 5 days (75 mg/kg, daily). HC toxin (Sigma-Aldrich, H7270) was injected (5 μg/kg, daily) as in previous studies (60).

### Tissue culture

HBMECs (ScienCell Research Laboratories, catalog 1000) were cultured per the manufacturer’s protocol (30). CRISPR/Cas9 genomic editing was used for MGP depletion. HBMECs were infected by lentiviral vectors, which contain gRNA for exon 1 of *Mgp* gene and Cas9 (Sigma-Aldrich). The infected cells were selected by puromycin. The positive clones were collected and expanded after 14 days of selection. The depletion of MGP was confirmed by real-time PCR. Transient transfections of HBMECs with Hdac2 siRNA (Silencer predesigned siRNA, Applied Biosystems) were optimized and performed as previously described (15). When compared with unrelated control siRNA and scrambled siRNA, the selected siRNAs resulted in a 90%–95% decrease in mRNA and protein levels, as determined by real-time PCR and immunoblotting, respectively. Lentiviral vectors containing CMV-Ezh1 were purchased from GeneCopoeia and applied to the cells per the manufacturer’s protocols.

### Isolation of cerebral ECs

Freshly harvested cerebral tissues from C57BL/6J mice were enzymatically dissociated with 250 μg/ml papain and 50 μg/ml DNase I in PBS/2% FBS at 37°C for 40 minutes. Cell suspension was washed and filtered with 70 μm strainers. After the myelin removal, cells were labeled with CD31-PE and CD45-FITC antibodies, and CD31^+^CD45^–^ cells were sorted by FACS (15).

### Micro-CT imaging

Micro-CT was performed by Scanco USA Inc. The perfusion with the MICROFIL compound and the preparation of the specimens were performed as previously described (15). All the samples were scanned on a high-resolution, volumetric micro-CT scanner (μCT 40, Scanco Medical). The image data were acquired with the following parameters: 10-μm isotropic voxel resolution; 200 ms exposure time; 2,000 views; and 5 frames per view. The micro-CT generated DICOM files were used to analyze the samples and to create volume renderings of the regions of interest (ROI). The raw data files were viewed using the MicroView 3D Volume Viewer and Analysis Tool (GE Healthcare) and AltaViewer software, version MIL-STD-1553. Additionally, images of the sample were generated using SCIRun (Scientific Computing and Imaging Institute).

### Vascular shunting

Fluorescent microspheres (15 μm, Invitrogen, F21010) were injected into the left cardiac ventricle immediately after sacrificing the mice, and the tissues were examined and photographed using a fluorescence dissection microscope.

### RNA analysis and RNA-Seq

#### RNA analysis.

Real-time PCR analysis was performed as previously described (61). GAPDH was used as a control gene (61). Primers and probes for mouse Mgp, Hdac1–11, Ezh1, Rbbp4, Ezh2, Eed, Suz12, Kdr, vWF, Snai 1, and Cdh2 were obtained from Applied Biosystems as part of TaqMan Gene Expression Assays.

#### RNA-Seq.

Three mice from each respective group were used for the isolation of CD31^+^CD45^–^ cells from freshly prepared cerebral cell suspensions as described above. Total RNA was isolated using the RNeasy Mini Kit (QIAGEN). RNA libraries were constructed using the KAPA Stranded mRNA-Seq Kit (Roche, KK8420), followed by sequencing conducted on the Illumina NovaSeq X Plus 10B Platform at the Technology Center for Genomics & Bioinformatics at UCLA. For the alignment of reads to the mouse genome build GRCm39, Spliced Transcripts Alignment to Reference (STAR, version 2.7.10a) was employed. Concurrently with the alignment, the quantification of reads per transcript was executed. Subsequently, the R package DESeq2 (version 1.36.0) was utilized to analyze RNA-Seq data. Initially, data were preprocessed to exclude genes with low counts, followed by data normalization and differential gene expression analysis. A log_2_ fold change threshold of 1 and an FDR of 0.05 were established as cut-offs for the identification of differentially expressed genes.

### Immunoblotting

Immunoblotting was performed as previously described (27). Equal amounts of tissue lysates were used for immunoblotting. Blots were incubated with specific antibodies to KDR, vWF, H4K8ac, H3, and H4 (Thermo Fisher Scientific, PA1-16613, PA5-104687, 710828, PA5-16183, and MA5-14816), SNAI1, CDH2, HDAC2, and EZH1 (Abcam, ab180714, ab76011, ab32117, ab289887), VE-cadherin, and H3K27me3 (Cell Signaling Technology, 2158 and 9733). β-Actin (Sigma-Aldrich, A2228) was used as a loading control. Densitometry was performed using ImageJ (NIH).

### Immunofluorescence

Immunofluorescence was performed as previously described (27). We used specific antibodies to HDAC2, EZH1, SNAI1, CDH2, SLUG, α-SMA, and NG2 (Abcam ab32117, ab289887, ab180714, ab76011, ab27568, ab7817, and ab255811), CD31 (Thermo Fisher Scientific, 14-0311-82), and VE-cadherin (BD Biosciences, 562243). The nuclei were stained with DAPI (Sigma-Aldrich, D9564).

### Specimens from human cerebral AVMs

Deidentified specimens were obtained from the Department of Pathology, David Geffen School of Medicine at UCLA. The specimens were not obtained specifically for this research, and none of the investigators involved in the research were able to ascertain the identity of the subjects. Sections of the specimens were subjected to immunofluorescence staining as previously described (27). Specific antibodies to HDAC2 and EZH1 (Abcam, ab32117 and ab289887) and CDH2 and PAR3 (Thermo Fisher Scientific, 33-3900 and PA5-45056) were used for the staining. ImageJ software was used to quantify the intensity of the immunofluorescence in microscopic images. Initially, images were segregated into individual channels, each rendered in grayscale (8 bit) format. Then ROIs were defined where the staining intensity would be measured. Following the optimization of the threshold, the images underwent analysis in accordance with the measurement settings, which included integrated intensity among others. The quantification of the integrated intensity at the lesion sites was then normalized to a control area, employing an identical scale factor, to determine the fold change in immunofluorescence intensity of target markers between lesion and control areas across 10 sections from each sample. Ten cases of deidentified human cerebral AVMs were used in this study. The correlation study was analyzed using Pearson’s correlation coefficient.

### Mouse surgery and cell transplantation

The murine model of hind-limb ischemia was developed as previously described (62). A 10 mm long incision in the skin was made toward the medial thigh. The femoral artery was exposed and separated from the femoral vein and nerve. Silk sutures were used to tie the proximal and distal ends of the femoral artery with double knots. CD31^+^CD45^–^CDH2^+^ or CD31^+^CD45^–^CDH2^–^ cells (5 × 10^5^) isolated from tamoxifen-injected *VE-cadherin^cre/ERT2^Mgp^fl/fl^* mouse cerebrum were transplanted into the surgical area, and the incision was closed. Laser Doppler perfusion imaging was used to monitor the blood flow at different time points. Histology and immunostaining were used to examine the vascularization after transplantation.

### Histone acetylation and methylation screening

CD31^+^CD45^–^ cerebral ECs were isolated from mice. Histone proteins were extracted by a histone extraction kit (Abcam, ab113476) and stored at –80°C for use. Histone proteins (200 ng) were used for each assay. Histone modifications were detected using the histone modification multiplex assay kits obtained from EPIGENTEK (P-3100-96 and P-3102-96). The procedures were performed per the manufacturer’s protocols. The specific histone modifications were detected with antibodies that were measured by a color development reagent. Individual histone modifications are proportional to the intensity of absorbance, which was measured by a plate reader at a wavelength of 450 nm. Total histone 3 or 4 was used as control.

### ChIP-Seq and ChIP assay

Specific antibodies were used to perform ChIP in order to enrich the genomic DNA from cerebral ECs as described before (61). Three samples from each group were pooled for library construction, and libraries were prepared according to the manufacturer’s instructions using the KAPA DNA HyperPrep Kit (Roche, KK8502). ChIP libraries were sequenced by the Technology Center for Genomics & Bioinformatics at UCLA. Reads from each sample were aligned to the mouse genome (mm10) using Bowtie2. The Homer tool was utilized to identify enriched peaks with 0.1% FDR and an enrichment of greater than 4-fold relative to the IgG control. Peak annotation was performed to associate peaks with nearby genes and to compute tag densities. The histogram of ChIP-fragment depth was derived using tag densities relative to the combined peaks from both groups. In the ChIP experiments, specific antibodies targeting H4K8ac (Thermo Fisher Scientific, 710828) and H3K27me3 (Abcam, ab6002) were utilized. For the ChIP assay, the primers for the promoters of Kdr and Snai1 were used as previously published (41, 42).

### Statistics

The analyses were performed using GraphPad InStat, version 8.0 (GraphPad Software). Data were analyzed using either unpaired, 2-tailed Student’s t *t*est or 1-way ANOVA with Tukey’s multiple-comparisons test for statistical significance.

### Study approval

The studies were reviewed and approved by the Institutional Review Board and conducted in accordance with the animal care guidelines set by UCLA. The investigation conformed to the National Research Council’s *Guide for the Care and Use of Laboratory Animals* (National Academies Press, 2011).

### Data availability

ChIP-Seq data were deposited in the Gene Expression Omnibus database (GEO GSE245171). RNA-Seq data were deposited in the GEO database (GSE262300). Values for all data points in graphs are reported in the Supporting Data Values file.

## Author contributions

Y Yao and KIB supervised the experiments, analyzed data, and wrote the manuscript. YZ, XW, Y Yang, LZ, XC, SC, AV, and JJ performed experiments and data analysis.

## Supplementary Material

Supplemental data

Unedited blot and gel images

Supporting data values

## Version 1 05/23/2024

In-Press Preview

## Version 2 08/01/2024

Electronic publication
