# Supplementary material for: Inhibition of endothelial histone deacetylase 2 shifts endothelial-mesenchymal transitions in cerebral arteriovenous malformation models
Source: J Clin Invest. 2024 May 23;134(15):e176758. doi: 10.1172/JCI176758 (PMC11290970; doi:10.1172/JCI176758)
Supplement: Supplemental data [file jci-134-176758-s036.pdf]

Inhibition of Endothelial Histone Deacetylase 2 Shifts Endothelial-Mesenchymal Transitions in Cerebral Arteriovenous Malformation Models

Yan Zhao, Ph.D.[1]*, Xiuju Wu, M.D., Ph.D.[1]*, Yang Yang, M.D., Ph.D.[1]*, Li Zhang, Ph.D.[1], Xinjiang Cai, M.D., Ph.D.[1], Sydney Chen, B.S.[1], Abigail Vera, B.S.[1], Jaden Ji, B.S.[1], Kristina I. Boström, M.D., Ph.D.[1, 2, 3], and Yucheng Yao, M.D., Ph.D.[1, 3]

[1] Division of Cardiology, David Geffen School of Medicine at UCLA, Los Angeles, CA 90095-1679, U.S.A.

[2] The Molecular Biology Institute at UCLA, Los Angeles, CA 90095-1570, U.S.A.

*Equal contribution

[3] To whom correspondence should be addressed:

Yucheng Yao, M.D. Ph.D. (lead contact); Kristina I. Boström, M.D., Ph.D.

Division of Cardiology, David Geffen School of Medicine at UCLA

Box 951679, Los Angeles, CA 90095-1679

Tel: 310-825-3239, Fax: 310-206-8553

E-mail: yyao@mednet.ucla.edu or kbostrom@mednet.ucla.edu

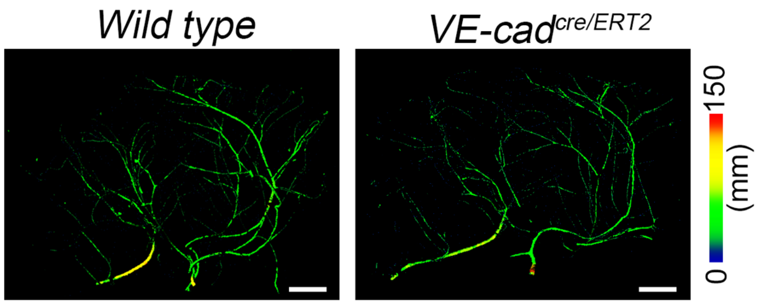

Supplemental Figure S1

Micro-CT imaging of wild type and VE-cadherin$^{cre/ERT2}$ mice with tamoxifen injection.

Activated Snai1	Independent compound																						Inactivated Snai1
339.9482	283.5370	323.5156	320.0423	319.2658	339.1712	322.8387	329.1841	337.1434	314.7994	337.2784	318.3127	341.0561	352.1839	330.7689	309.4726	320.8978	299.6902	316.0641	326.6843	321.4144	330.2672	331.7101	24.3637
348.8233	331.8138	353.0361	321.1388	338.5302	324.9264	326.3737	320.3897	325.7608	322.7522	318.8831	332.5648	317.6454	326.2438	321.4900	318.2944	333.9698	319.2480	312.8644	335.7097	327.2361	310.7715	327.7574	34.6074
353.4205	332.2623	328.2005	318.7766	325.4468	325.0413	323.2277	313.8918	310.9538	316.0255	324.2983	320.8155	322.3865	318.0473	312.4231	303.8310	325.8436	315.3327	333.1608	327.9415	316.3968	315.5872	317.1495	43.6261
347.3846	330.9077	323.6816	324.1082	332.9455	323.2959	323.9694	365.9138	323.2428	316.1196	319.8519	318.3161	327.2636	317.5969	341.8532	333.9634	320.5163	307.2141	316.7248	339.9518	327.3884	318.3051	323.1139	33.9391
354.1666	332.1008	320.2708	327.0538	323.5739	320.4033	310.6138	326.2853	322.9112	319.1384	317.0486	327.8045	334.3002	327.3511	317.1831	327.9630	248.6350	311.5184	334.1249	309.7899	336.8532	328.0541	318.6379	43.0705
360.4470	321.5040	316.8941	318.2272	344.5025	309.9136	313.3772	314.6861	336.0274	326.6807	319.1311	322.4761	320.1853	323.5587	* 34.7768	320.6520	321.3429	319.5539	331.3681	331.4874	320.5424	332.3518	322.5947	38.9286
351.6304	321.4676	330.9426	325.1324	320.7143	321.8349	337.4077	323.9597	327.4470	328.8337	317.1691	329.1943	328.5476	329.8268	312.9420	308.4222	320.7876	320.1346	321.0126	337.7376	330.0355	320.9746	328.7085	40.2384
341.3753	333.8876	319.9263	312.2554	321.9034	318.5967	320.6778	315.5305	327.7511	321.6970	330.9672	323.1543	317.7736	327.4431	307.0682	324.2638	321.5585	320.9885	328.4014	341.2357	314.1849	310.2999	335.1769	39.4588
332.3551	339.3044	318.1175	320.0853	324.4487	318.4532	319.1221	329.4369	310.7704	323.3996	334.3271	274.0007	315.7166	330.9982	319.0500	317.1888	329.3977	339.7642	319.4662	322.4916	325.8906	313.7724	325.1904	45.7331
335.5417	330.6268	325.4139	333.8625	325.4631	321.3950	316.5108	299.2034	309.1760	312.5683	310.3817	314.3239	332.8176	329.8848	315.9303	317.3716	320.8934	333.6879	331.3007	311.3401	313.6141	326.0093	321.1018	47.9272
351.1190	323.6530	313.9511	318.3164	326.5205	324.8589	320.5033	351.4052	316.7990	320.5007	327.1899	322.5138	317.4155	329.7774	336.4242	325.3459	330.1408	316.7448	323.8758	337.3058	314.5974	322.4558	311.8156	37.9958
352.9757	336.0399	326.3870	311.8036	322.9938	317.4816	308.9198	323.1920	321.4255	323.8575	315.4744	313.1173	328.5075	314.9607	323.0546	311.8661	325.5503	318.3502	320.4430	317.1734	313.4104	315.9539	326.4998	30.1361
335.7285	321.6567	320.5460	333.6260	324.4978	318.1482	312.3476	318.1251	310.5165	325.1432	325.1739	326.1084	315.2314	327.3649	336.6576	314.4740	324.4502	324.6146	323.7812	323.3783	328.8527	222.1533	310.7977	35.0380
342.2017	334.8776	354.1143	319.0226	315.4612	315.6131	324.1544	320.4689	331.0269	329.4576	321.4679	311.7760	334.3513	317.9085	311.2857	347.0239	322.1369	316.2576	311.7477	314.9386	309.2548	318.8706	320.0590	23.2896
347.4648	334.7757	327.9123	321.1269	318.4898	314.1931	316.9266	315.1817	318.4524	326.8603	326.1614	312.6928	323.7726	326.0925	314.4015	312.2283	332.5499	316.7825	315.0587	315.6396	320.7440	312.1197	313.8951	33.3786
369.9179	284.4419	316.0998	343.9060	326.9169	332.5773	331.2875	323.6738	314.7110	315.7801	332.1148	329.4448	330.4068	342.4033	316.4610	324.4127	335.2259	319.1392	324.1424	323.5040	321.5917	321.1864	325.3391	18.9779

Supplemental Figure S2

Compound screening for reducing Snai1 expression. Star, GFP level of HC-toxin treatment.

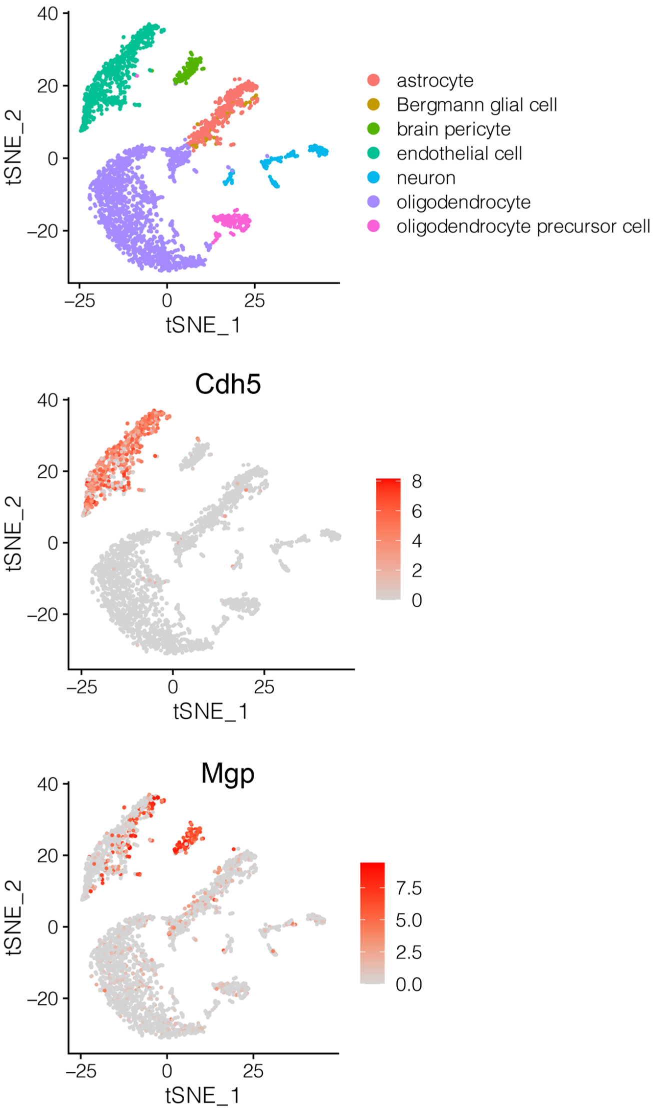

Supplemental Figure S3

MGP expression in cell clusters shown by single-cell RNA sequencing.
