# Supplementary figures and images for: Inhibition of endothelial histone deacetylase 2 shifts endothelial-mesenchymal transitions in cerebral arteriovenous malformation models
Source: J Clin Invest. 2024 May 23;134(15):e176758. doi: 10.1172/JCI176758 (PMC11290970; doi:10.1172/JCI176758)

Figure 2B

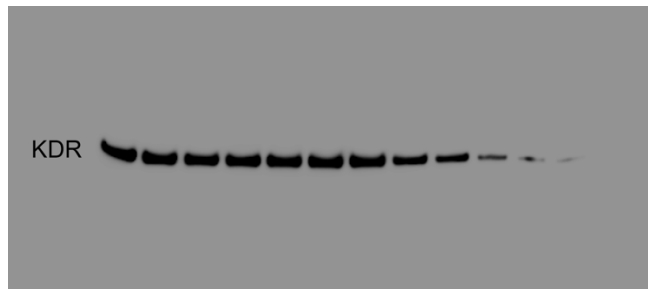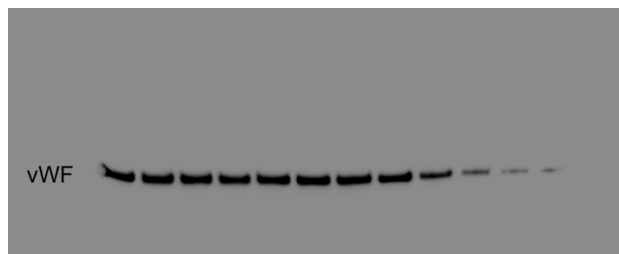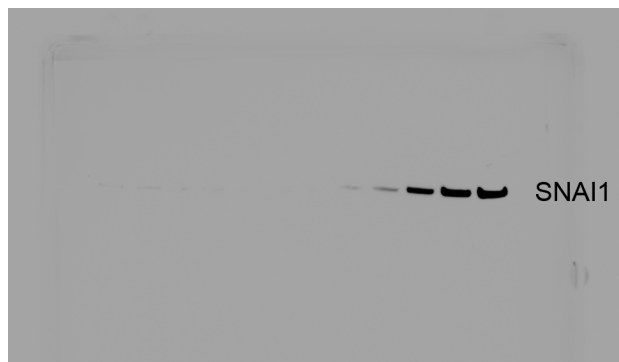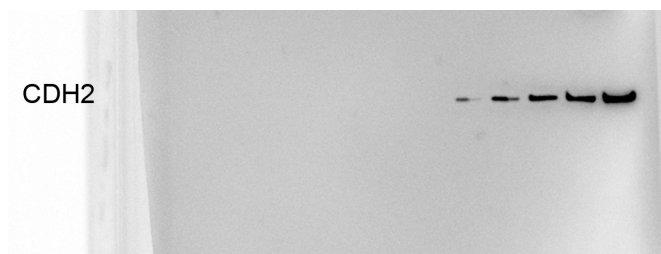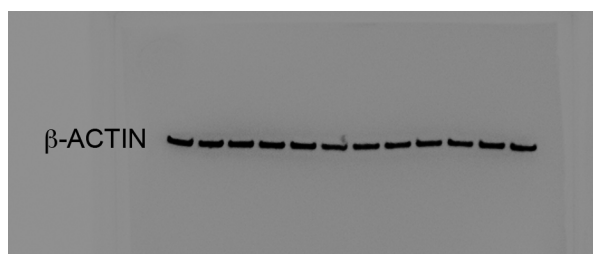

Figure 5B

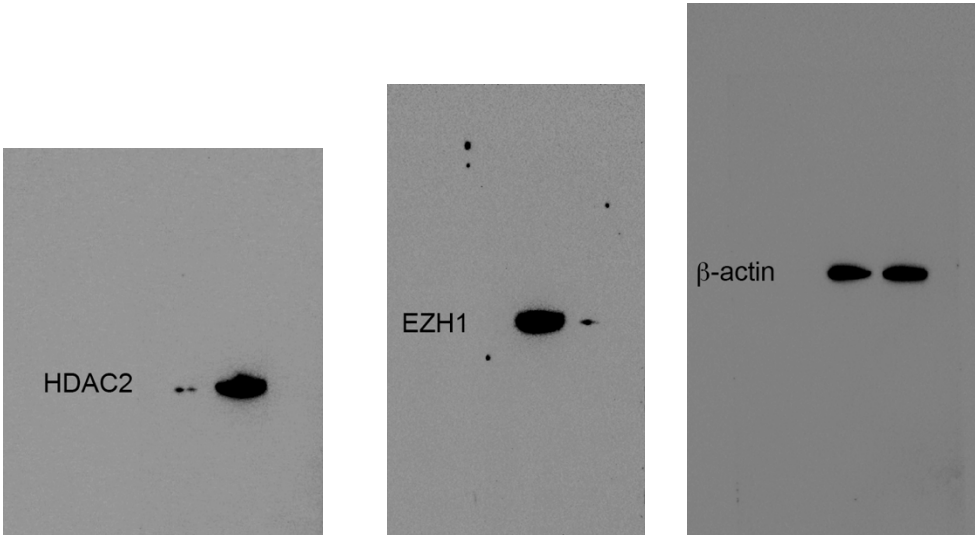

Figure 6B

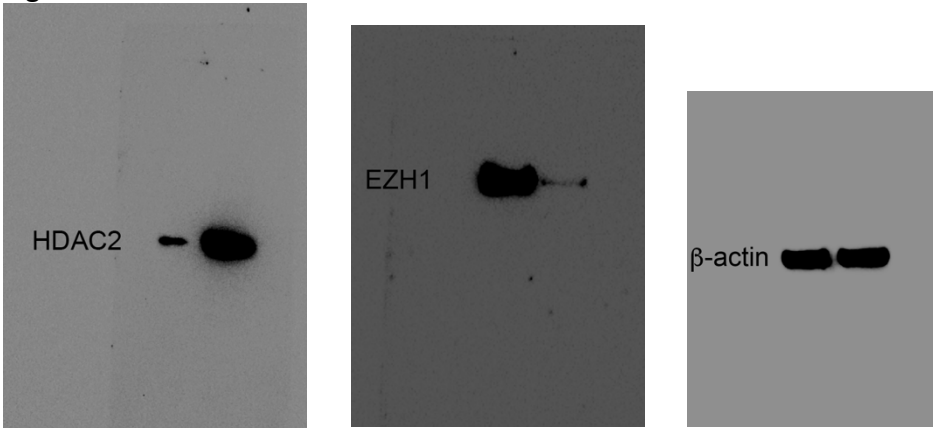

Figure 8B

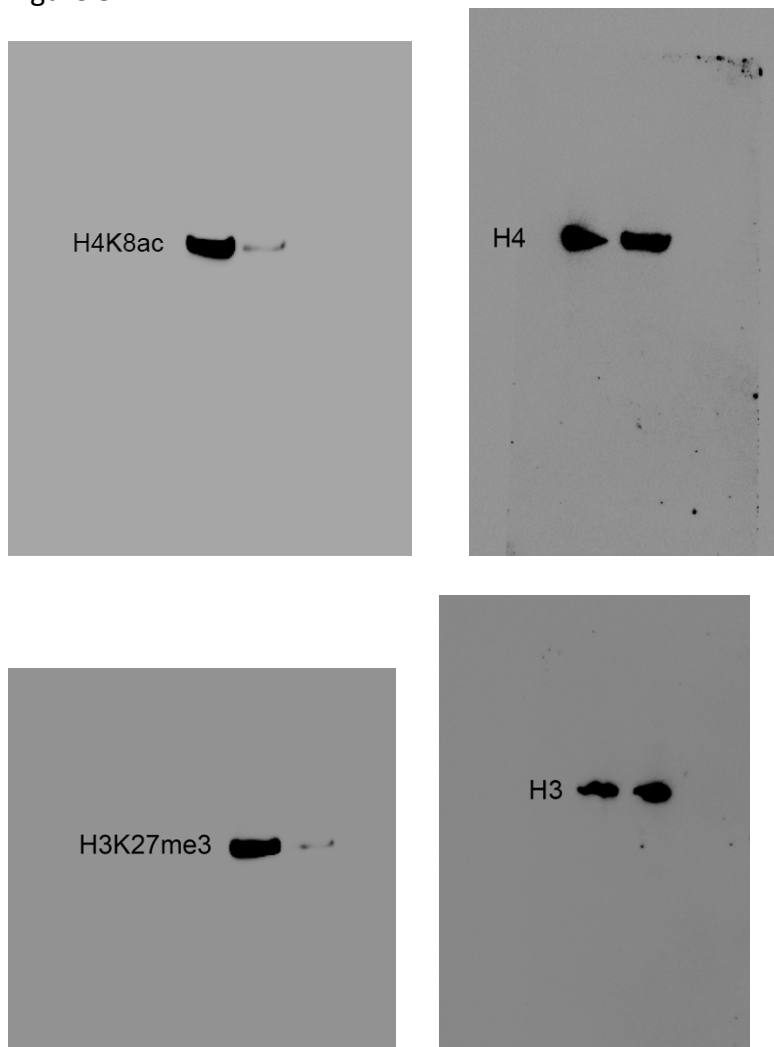

Figure 8C

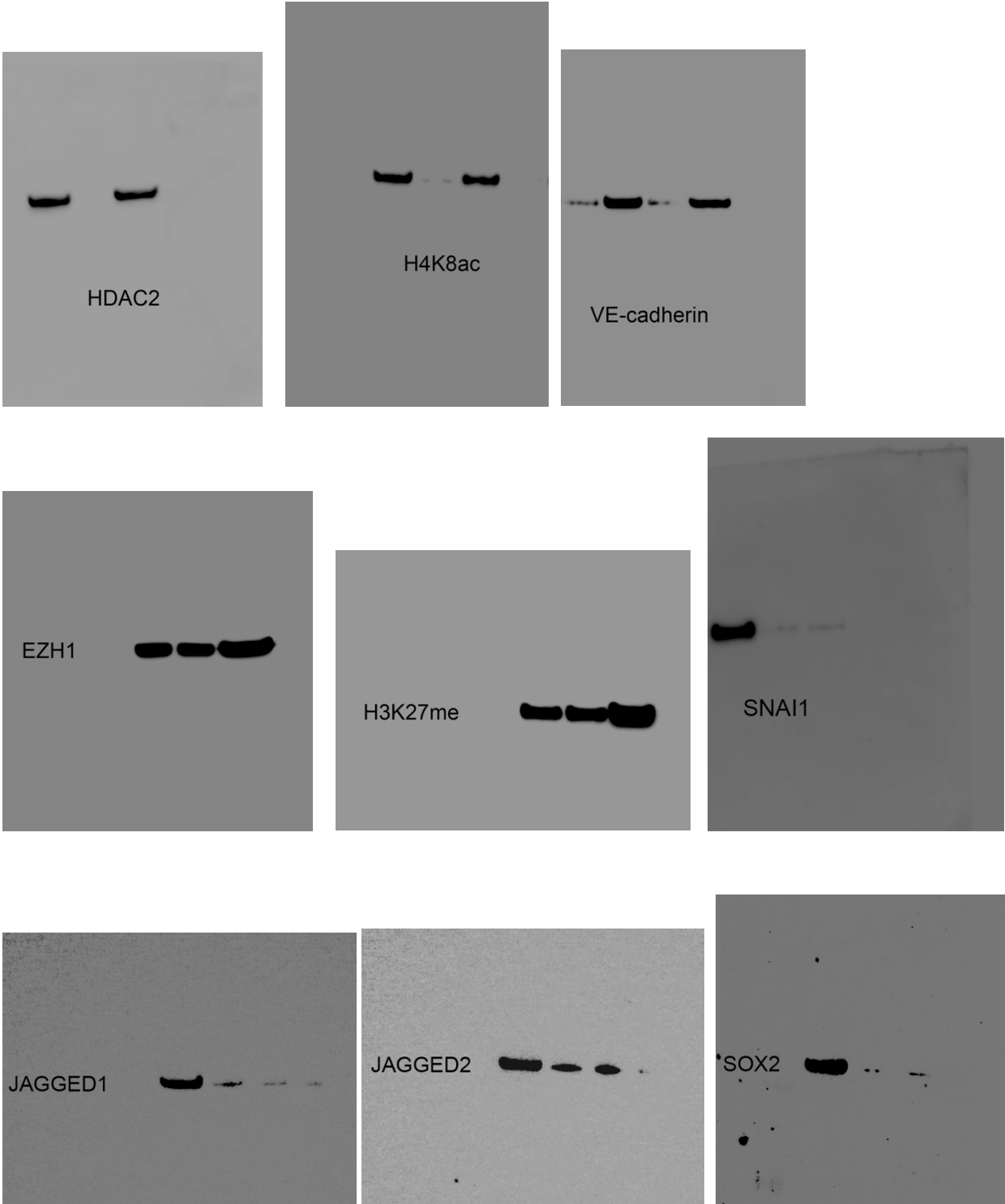

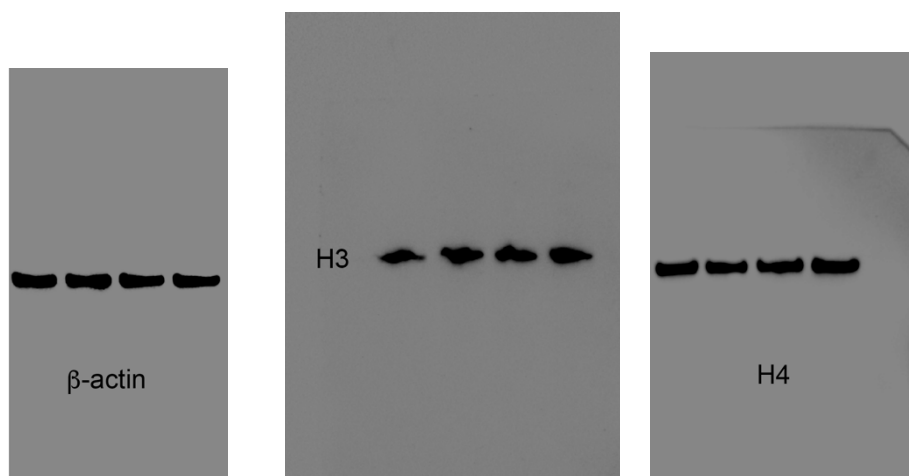

Figure 8F

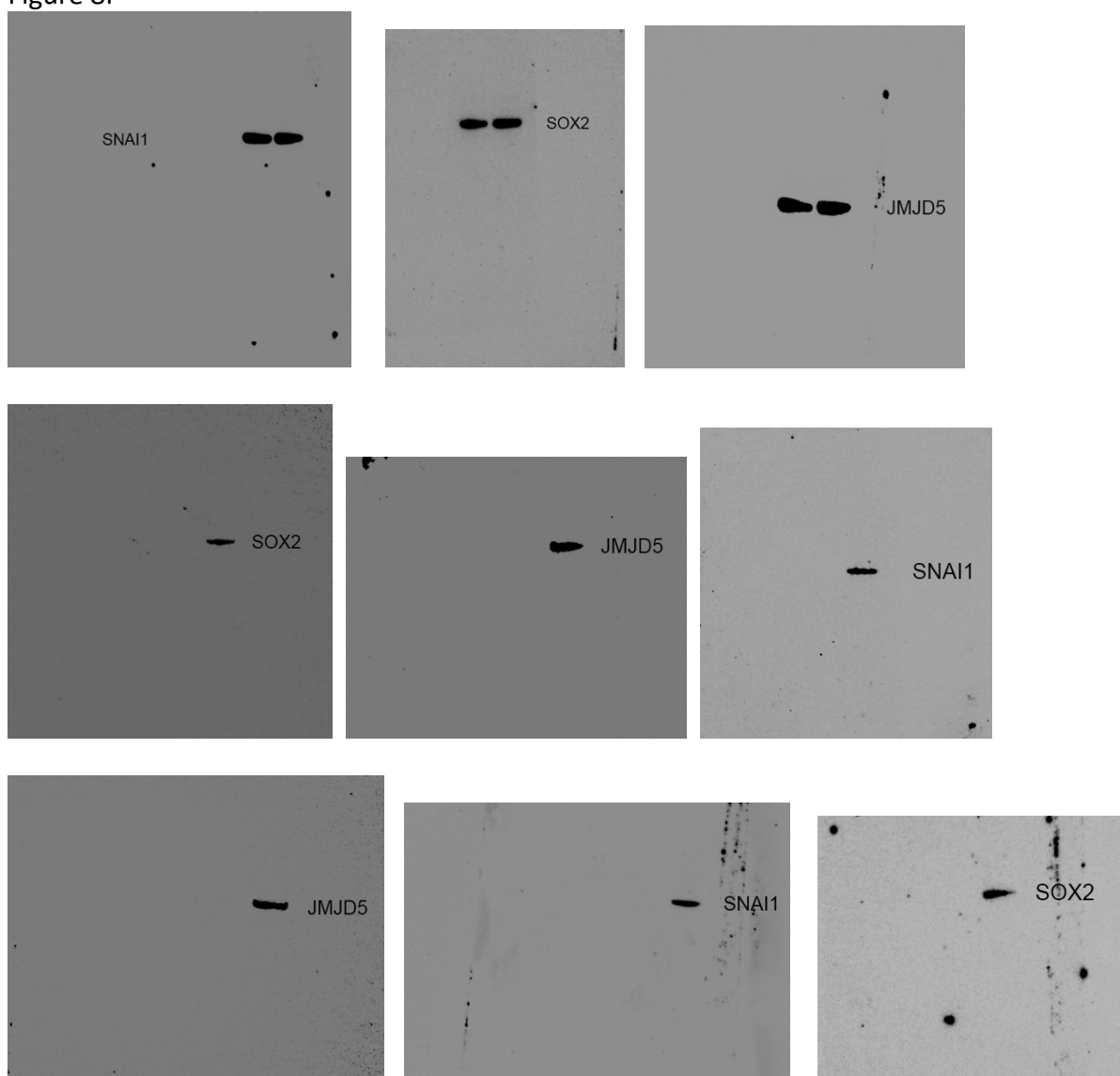

Supplement: Unedited blot and gel images [file jci-134-176758-s037.pdf]
